# Combined Acquisition Technique (CAT) for Neuroimaging of Multiple Sclerosis at Low Specific Absorption Rates (SAR)

**DOI:** 10.1371/journal.pone.0091030

**Published:** 2014-03-07

**Authors:** Armin Biller, Morwan Choli, Martin Blaimer, Felix A. Breuer, Peter M. Jakob, Andreas J. Bartsch

**Affiliations:** 1 Department of Neuroradiology, University of Heidelberg, Heidelberg, Germany; 2 Research Center for Magnetic-Resonance-Bavaria (MRB), Wuerzburg, Germany; 3 Department of Experimental Physics 5, University of Wuerzburg, Wuerzburg, Germany; 4 FMRIB Centre, University of Oxford, Oxford, United Kingdom; Julius-Maximilians-Universität Würzburg, Germany

## Abstract

**Purpose:**

To compare a novel combined acquisition technique (CAT) of turbo-spin-echo (TSE) and echo-planar-imaging (EPI) with conventional TSE. CAT reduces the electromagnetic energy load transmitted for spin excitation. This radiofrequency (RF) burden is limited by the specific absorption rate (SAR) for patient safety. SAR limits restrict high-field MRI applications, in particular.

**Material and Methods:**

The study was approved by the local Medical Ethics Committee. Written informed consent was obtained from all participants. T2- and PD-weighted brain images of n = 40 Multiple Sclerosis (MS) patients were acquired by CAT and TSE at 3 Tesla. Lesions were recorded by two blinded, board-certificated neuroradiologists. Diagnostic equivalence of CAT and TSE to detect MS lesions was evaluated along with their SAR, sound pressure level (SPL) and sensations of acoustic noise, heating, vibration and peripheral nerve stimulation.

**Results:**

Every MS lesion revealed on TSE was detected by CAT according to both raters (Cohen’s kappa of within-rater/across-CAT/TSE lesion detection κ_CAT_ = 1.00, at an inter-rater lesion detection agreement of κ_LES_ = 0.82). CAT reduced the SAR burden significantly compared to TSE (p<0.001). Mean SAR differences between TSE and CAT were 29.0 (±5.7) % for the T2-contrast and 32.7 (±21.9) % for the PD-contrast (expressed as percentages of the effective SAR limit of 3.2 W/kg for head examinations). Average SPL of CAT was no louder than during TSE. Sensations of CAT- vs. TSE-induced heating, noise and scanning vibrations did not differ.

**Conclusion:**

T2−/PD-CAT is diagnostically equivalent to TSE for MS lesion detection yet substantially reduces the RF exposure. Such SAR reduction facilitates high-field MRI applications at 3 Tesla or above and corresponding protocol standardizations but CAT can also be used to scan faster, at higher resolution or with more slices. According to our data, CAT is no more uncomfortable than TSE scanning.

## Introduction

High-field MRI at 3 Tesla and beyond promises unprecedented signal-to-noise ratios (SNR), image resolutions and acquisition speed. A major drawback is the raised radiofrequency (RF) power deposition which is monitored by the specific absorption rate (SAR). Doubling the static field strength from 1.5 to 3 Tesla, for example, quadruples the SAR. Thus, MRI at high fields is particularly restricted by SAR safety limits. The higher the static magnetic field the more patients experience unpleasant RF-induced heating during MRI [1]–[3]. SAR limits constitute a problem especially for fast spin-echo (FSE) based sequences, such as turbo spin-echo (TSE) or rapid acquisitions with relaxation enhancement (RARE) [4]. These are frequently used to obtain T2- and proton density (PD-) contrasts to determine, for instance, the lesion load in Multiple Sclerosis (MS). Since each 180° RF excitation transmits four times the energy of a 90° pulse, TSE sequences with high turbo factors are very SAR-intensive. SAR monitoring at 3 Tesla and beyond often requires unwanted protocol adjustments to individuals of different body weights which are detrimental to standardized image acquisition across subjects in controlled studies and trials. Echo-planar-imaging (EPI), on the contrary, does not depend on such refocusing pulses and is *per se* very economical in terms of the associated SAR. Combined acquisition techniques (CAT) by hybrid pulse sequences of TSE and EPI reduce the SAR [5], [6]. A recent study investigating benefits of T2-/PD-CAT for neuroimaging at higher magnetic fields [7] showed that the hybrid EPI/TSE-combination in CAT can achieve substantial SAR reductions at equivalent image quality. However, clinical effectiveness and diagnostic equivalence of CAT and TSE have not yet been demonstrated. Given that SNR is slightly lower for CAT compared to TSE [7], [8], this is essential prior to translating CAT into clinical applications and practice.

Here, we compare the diagnostic equivalence, SAR, patient comfort and artifacts of CAT and TSE in the first clinical CAT application to MS. Theoretically, the EPI module of CAT could introduce local signal loss and geometric distortions in the phase-encoding direction in areas of local field inhomogeneities from susceptibility gradients in neighbouring tissues, especially at the skull base. It may also increase acoustic noise levels due to its rapid read-out gradient switches [9], [10]. Therefore, we assess image artifacts, record the sound and collect ratings of acoustic noise during CAT and TSE. Furthermore, rapidly alternating EPI read-outs are prone to evoke muscle twitches due to peripheral nerve stimulation [11]. These may lead susceptible patients to interrupt the MRI exam. The frequency of such unwanted events is recorded and a strategy to avoid peripheral nerve stimulation during CAT is developed.

## Materials and Methods

### Ethics Statement

The study was approved by the local Medical Ethics Committee (Faculty of Clinical Medicine, University of Wuerzburg, Germany), and all participants gave written informed consent prior to enrolment. The procedures that followed were in accordance with the declaration of Helsinki.

### Imaging Sequences

All measurements were performed on a 3 Tesla TimTrio MR system (Siemens Medical, Erlangen, Germany) using a 12-channel phased-array head coil. The order of CAT and TSE scanning was varied by rotation according to a Latin square.

**Figure 1:**
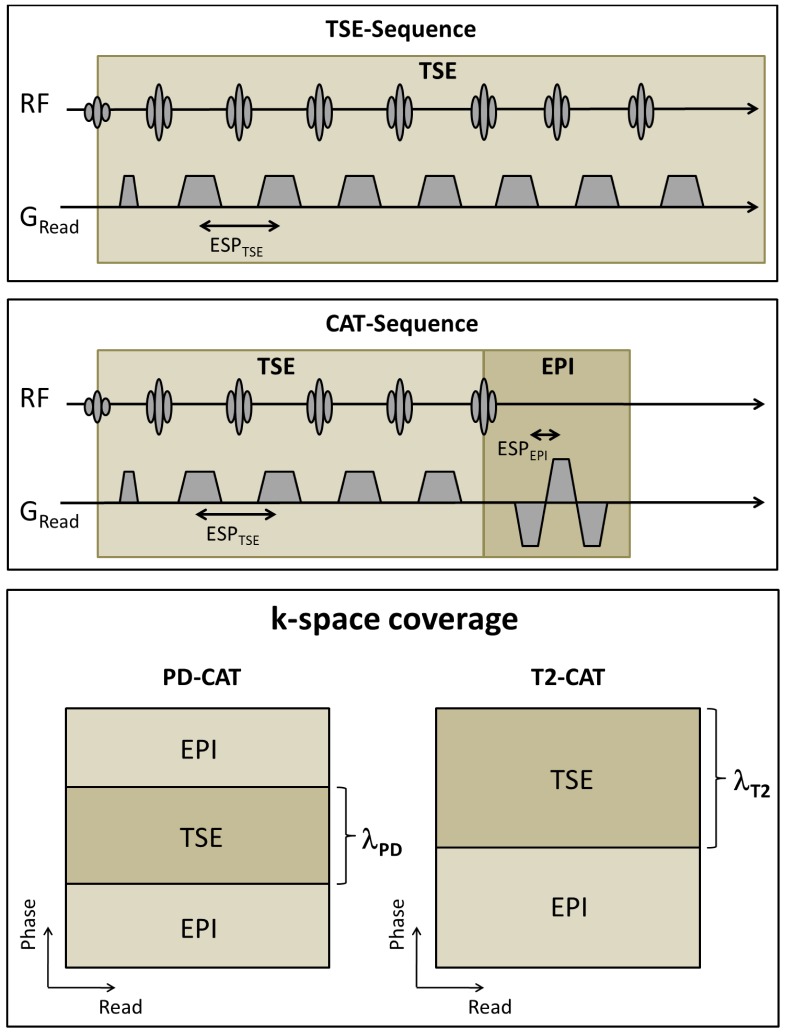
MR pulse sequence schemata of conventional TSE (A) and CAT imaging (B) compared to each other in this study. The k-space coverage of PD- and T2-CAT is shown in (C).

In figure 1, the pulse sequence schemata of TSE and CAT and the k-space coverage of T2- and PD-weighted CAT images are illustrated. In order to maintain comparable contrasts, the echo (TE), repetition (TR) and acquisition (TA) times of the TSE and CAT sequences was set to identical values. Table 1 summarizes the parameter settings.

**Table 1:** CAT and TSE sequence parameter.

		CAT	TSE
**FOV (mm^2^)**	T2	172×230	172×230
	PD	230×230	230×230
**slice thickness (mm)**		2.5	2.5
**number of slices**	T2	42	42
	PD	30	30
**phase encoding**	T2	R>>L	R>>L
	PD	A>>P	A>>P
**interslice gap (%)**		15	15
**matrix size**		288×320	288×320
**turbo factor**		13	13*
**TR (ms)**		8000	8000
**TE (ms)**	T2	84	84
	PD	10	10
**ESP (ms)**	T2	2.6§	12
	PD	2.6§	9.7
**band width (Hz/Px)**	T2	500§	250
	PD	500§	320#
**flip angle (°)**		90	90
**refocusing angle (°)**	T2	180	180
	PD	180	180
**CAT factor λ** ¶	T2	0.54	n.a.
	PD	0.4	n.a.
**Acquisition time (secs)**	T2	160	160
	PD	182	182

*Reduced to 9 in 4 patients in whom TF = 13 already exceeded the SAR limits in TSE imaging.

# BW of PD was set higher than for T2 in order to achieve low TE values minimizing the T2-effect.

¶ At λ = 0.5, half of k-space lines are read out by the TSE module.

§ Of the EPI module.

Fat suppression was applied to minimize artifacts, esp. from EPI in CAT.

### Patients

N = 40 patients (28 female, 12 male; 39.4±17.1 years old) suffering from Multiple Sclerosis (MS) diagnosed according to the revised McDonald Criteria [12] were measured using TSE and CAT sequences each with T2- and PD-contrast (hereafter, T2-/PD-CAT). One patient was measured twice upon a 6 months follow-up. Exclusion criteria were neurologic illnesses other than MS, any other medical illness and claustrophobia.

### Evaluation of Multiple Sclerosis (MS) Lesion Load

Demyelinating MS lesions were recorded on TSE- and CAT-based axial T2- and sagittal PD-weighted images by two blinded, experienced and board-certificated neuroradiologists (A.B. and A.J.B.). CAT and TSE images were read in an automatically assigned random order, and in half of the patients CAT was read first by both raters. The inter-rater agreement was measured by Cohen’s kappa coefficients in terms of the across-rater lesion detection (κ_LES_). Additionally, the within-rater lesion detection agreement across TSE and CAT was obtained by a second Cohen’s kappa coefficient (κ_CAT_). Finally, for each contrast (T2 and PD), the lesion load detected in the TSE- and CAT-based MR images was compared on a pairwise basis using a two-sided, one-sample paired t-test (see below).

### Specific Absorption Rate (SAR) and other Measurements/Ratings

For each patient, the actual weight was recorded. The corresponding SAR values were then automatically calculated by the MR system. SAR values are expressed as percentages of the SAR limit of 3.2 W/kg for head examinations according to the effective IEC regulations [13]. Any instance of peripheral nerve stimulation with involuntary muscle twitches occurring despite automatic stimulation monitoring was recorded.

**Figure 2:**
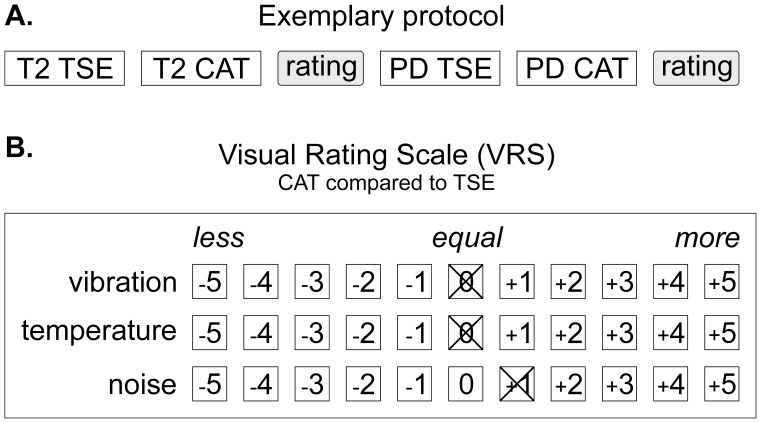
Sound waves and frequency spectra for T2-weighted TSE, CAT (λ = 0.5) and pure EPI. Peak SPLs increase the higher the EPI proportion (i.e., the lower the CAT factor λ) but average SPLs of CAT at λ = 0.5 and TSE are comparable (*top*). EPI read-outs introduce a fundamental frequency peak at the reciprocal of twice the echo spacing (here: ESP = 2.6 ms/FFT peak = 192 Hz) which increases the higher the EPI proportion (i.e., the lower λ is set; *bottom*).

Calibrated sound waves (SW) and average sound pressure levels (SPL) were measured at the patient’s ears by headphones with built-in optical microphones of 0.1 dB SPL accuracy (OptoActive, Optoacoustics Ltd., Moshav Mazor, Israel). Passive noise attenuation of the headphones amounted to 29 dB (Noise Reduction Rating according to the Environmental Protection Agency, NRR/EPA). SWs were recorded using Audacity v. 2.0.3 (http://audacity.sourceforge.net/) at a sampling rate of 96 kHz, with spectral frequency analysis being performed by a Fast Fourier Transform (FFT) using a Welch window. SW and FFT were plotted using Matlab (R2011a, The Mathworks, Inc., Natick, MA, USA; cf. Fig. 2).

**Figure 3:**
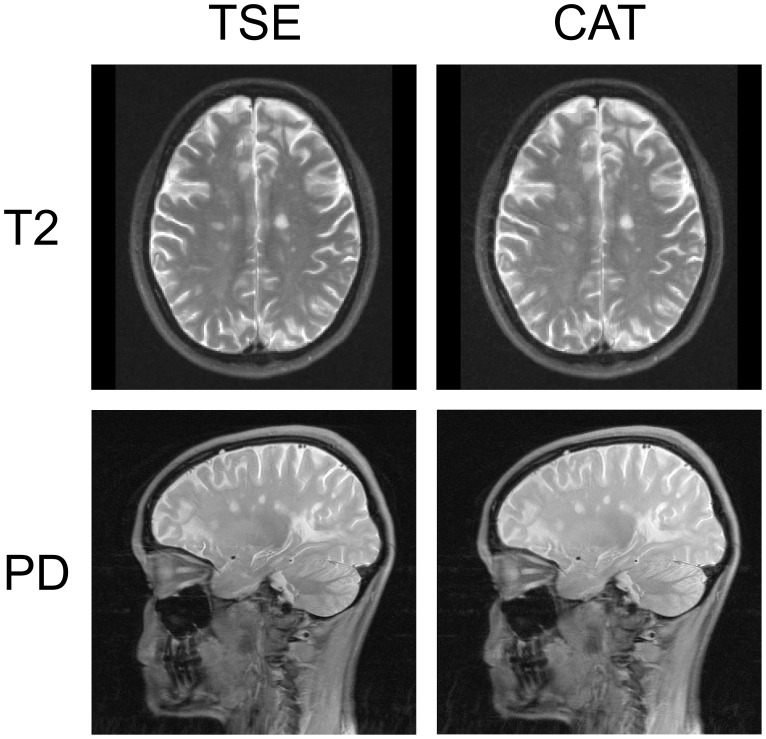
Measurement protocol and Visual Rating Scale (VRS). The order of CAT and TSE sequences was varied by rotation according to a Latin square. Upon measurement of a CAT/TSE double (**A**) the patient rated the two sequences in comparison to each other. Ratings scored sensations of temperature (RF-induced heating), acoustic noise and scan vibrations (**B**). Negative VRS values indicate less heating, acoustic noise and scan vibrations during CAT vs. TSE imaging, positive values indicate that higher temperatures, acoustic noise and vibration levels were perceived during CAT vs. TSE imaging while zero refers to no subjective difference between the CAT and TSE.

Subjective sensations of RF-induced heating, acoustic noise and mechanical vibrations during the scanning were rated using a 11-point visual rating scale (VRS) directly comparing TSE and CAT against each other upon completion of each corresponding sequence set (cf. Fig. 3).

### Image Processing and Statistical Analysis

**Figure 4:**
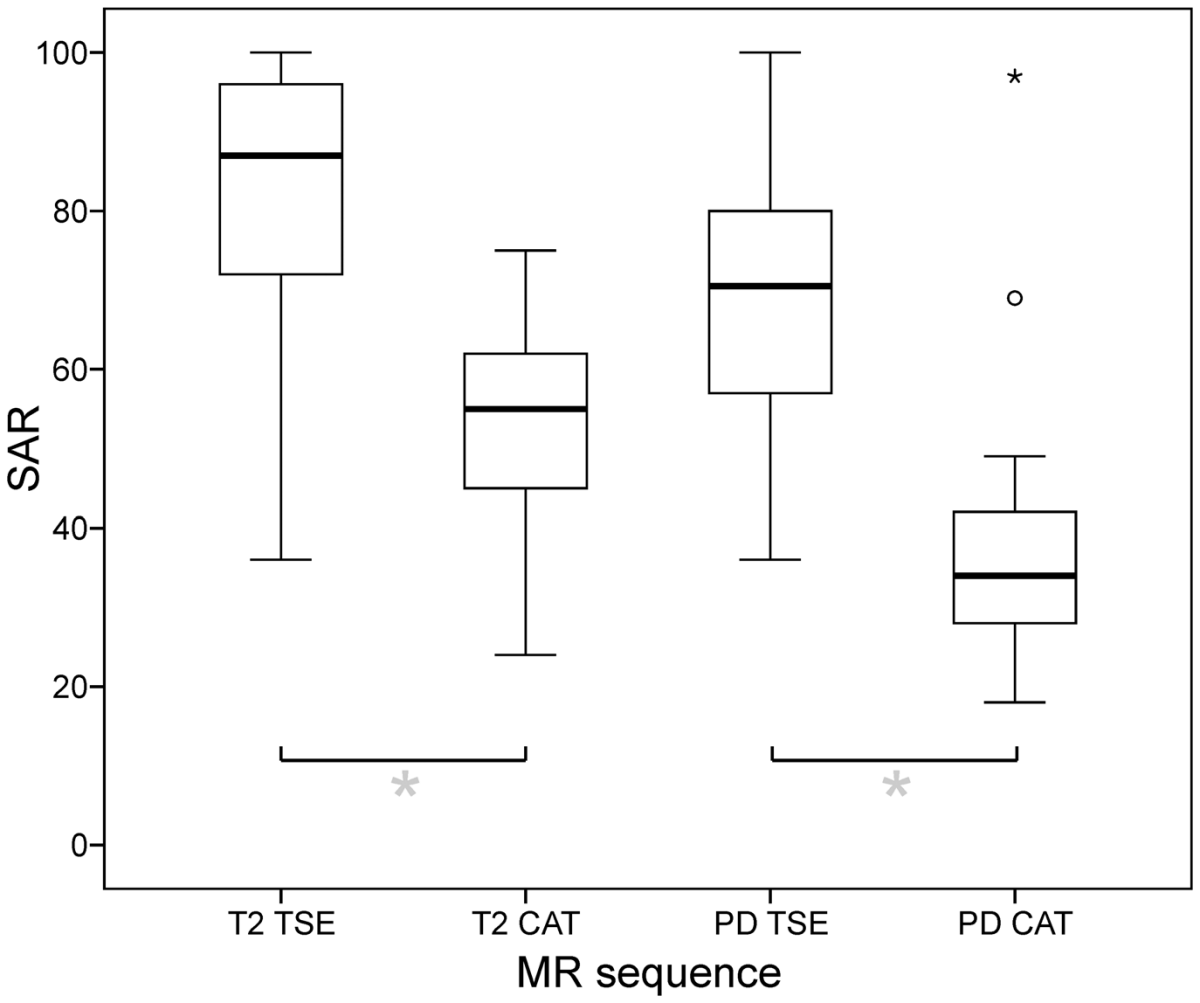
TSE and CAT brain images in Multiple Sclerosis (MS). Exemplary T2- (upper row) and PD-weighted images acquired by TSE (*left column*) and CAT (*right column*) sequences are shown. The data from this representative patient illustrate, along with those from another in Fig. 5, the diagnostic equivalence of both MR techniques: Every lesion picked up on the TSE image is detected on the CAT image as well. Minimally reduced SNR of CAT compared to TSE which has previously been quantified [7] is noticeable upon close visual inspection but does not impede diagnostic accuracy (spatial noise ratio of TSE to CAT was 0.82 for T2- and 0.88 for PD-weighted images).

**Figure 5:**
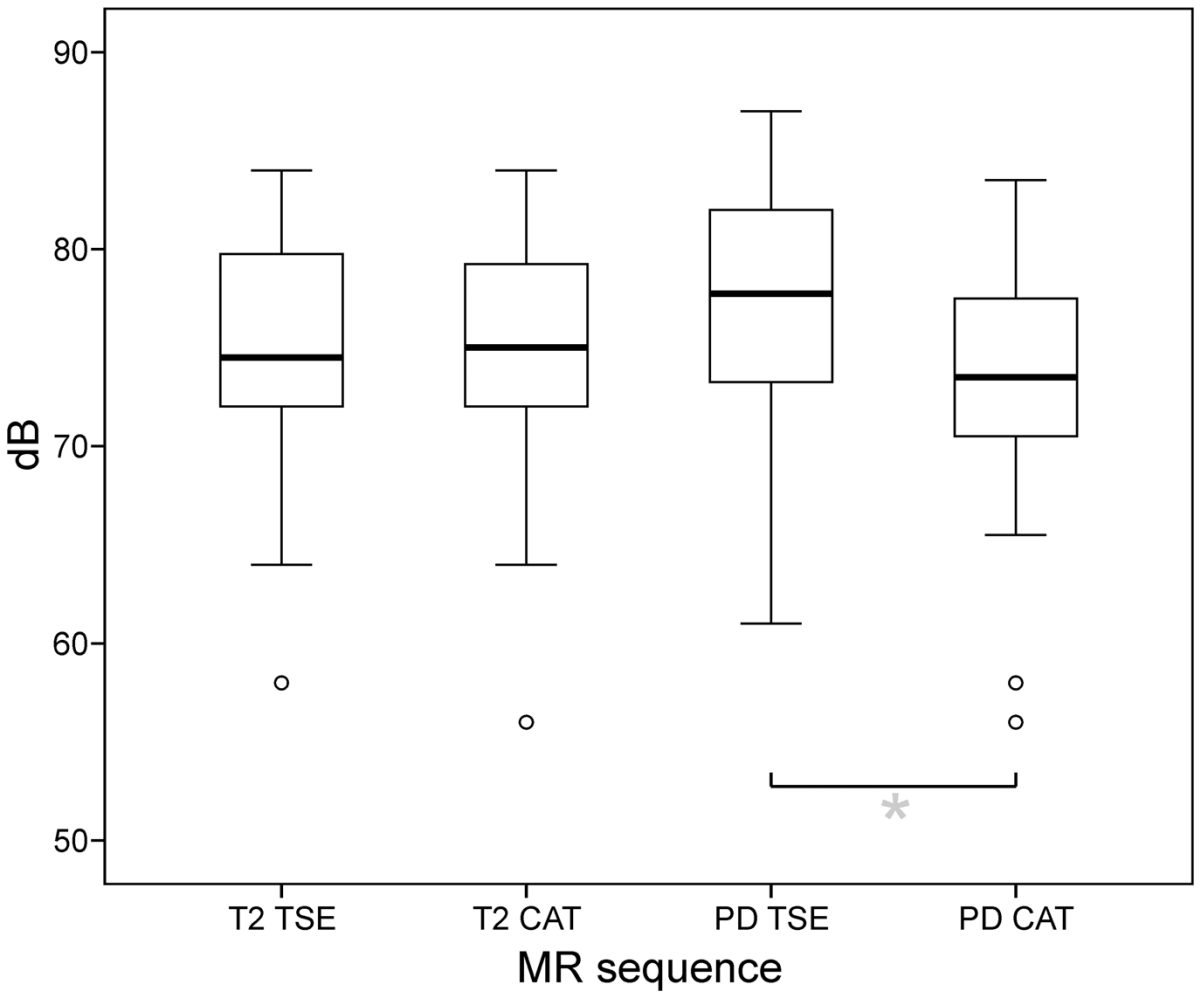
Image artifacts and distortions: CAT artifacts and distortions in phase-encoding direction (here from right to left, R>>L) as detected at the skull base level. The straight gyrus and olfactory sulcus are slightly displaced leftwards in CAT (*top right*; depending on blip polarity, cf. [7]) compared to TSE (*top left*). Otherwise, CAT contours (red outlines in *lower left*) overlay almost perfectly with TSE (*lower left*) and vice versa (*lower right*) upon CAT/TSE co-registration (RMS deviation ≤1.6e^−6^ mm). Artifacts did not significantly interfere with MS lesion detection. Even tiny multiple T2-hyperintense demyelinations (*arrows*) are well visualized despite minimal blurring (at a spatial noise ratio TSE/CAT of 0.81 in this case).

Spatial noise (N) was estimated for images of two representative patients (cf. Fig. 4 and 5) by the standard deviation of the signal outside the head/neck using the Brain Extraction Tool (BET2) [14] (to generate a binary brain mask), fslmaths (to dilate this mask), manual editing in fslview (to obtain a slightly overinclusive full head/neck mask), fslmaths (to get its inverse), and fslstats, all part of the FMRIB Software Library (FSL 5.0.2.2, http://fsl.fmrib.ox.ac.uk/fsl/fslwiki/) [15]. Spatial noise ratios of TSE to CAT are reported.

In order to assess artifacts and distortions, images of all patients were inspected and CAT/TSE volumes of one representative patient were registered to each other using the inversely consistent, rigid body registration with 6 degrees of freedom (DoF) provided by mri_robust_register [16] (part of FreeSurfer v. 5.3.0, http://surfer.nmr.mgh.harvard.edu/fswiki). Contour overlays of CAT on TSE and vice versa were generated for one representative patient using slicer (part of FSL; cf. Fig. 5, bottom). The root mean square (RMS) deviation between the identity transformation and residual misalignments of the co-registered images as estimated by a 6 DoF transformation using FMRIB’s Linear Image Registration Tool (FLIRT, part of FSL) [17], [18] was calculated using rmsdiff (also part of FSL).

Statistical testing was performed using SPSS Statistics (Version 21, IBM). SAR as well as acoustic noise measurements of TSE and CAT sequences were compared by one-sample paired t-tests. SAR values were analyzed for differences between CAT and TSE by one-sided testing (due to the known SAR advantage of CAT [7]) while average SPL values and subjective ratings of TSE and CAT with respect to temperature, acoustic noise and vibration were compared by two-sided testing. The standard significance level α was 0.05, corresponding to a type I error probability ≤5%.

## Results

### Multiple Sclerosis (MS) Lesion Detection

For all patients, every hyperintense demyelinating MS lesion recorded on axial T2- and sagittal PD-TSE images was also detected in the corresponding CAT images by both raters (A.B. and A.J.B.) who were blinded to the actually used sequence (TSE vs. CAT) upon their assessment (κ_CAT_ = 1.00, at an inter-rater lesion detection agreement of κ_LES_ = 0.82). Notably, this was also the case when CAT was read first. Thus, irrespective of the order of reading there was no difference in the number of lesions recorded on TSE and CAT (p = 1.00). Given that it is the count (and not the size) of demyelinating lesions that is diagnostic according to all currently used and proposed MRI criteria for MS, diagnostic equivalence of CAT vs. TSE is hereby established. Furthermore, the T2-signal characteristics of MS lesions were considered to be generally identical on TSE and CAT images by both raters. T2- and PD-weighted example images of representative cases are shown in Figures 4 and 5. Due to minor SNR reductions, which have previously been demonstrated for CAT [7], and the slightly increased spatial noise within CAT images (cf. *Image noise, artifacts and distortions* section), small MS lesions may appear marginally blurred (Fig. 5). However, both raters considered general lesion conspicuity to be equal on CAT vs. TSE and other important differences (e.g., in the appearance of flow void, other signal loss etc.) were not noted (cf. Figs. 4 and 5).

### Specific Absorption Rate (SAR)

**Figure 6:**
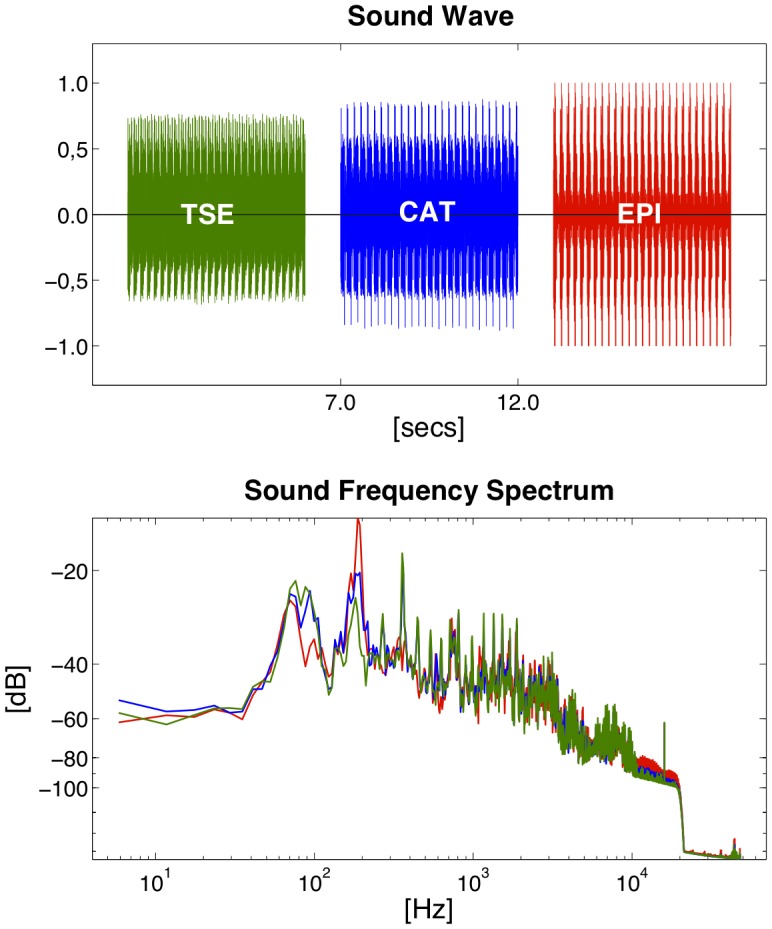
Specific absorption rate (SAR) of TSE and CAT sequences. Relative SAR reductions of CAT compared to TSE sequences was significant for both, the T2- (29.0±5.7%; p<0.001) and the PD -contrast (32.7±21.8%; p<0.001). (*box*: upper and lower quartiles, *thick black line*: median, *whiskers*: most extreme values of the interquartile range, *circle*: outlier, *asterisk*: extreme value; SAR values expressed as percentages of the effective SAR limit of 3.2 W/kg for head examinations according to IEC).

On average, SAR was 82.6 (±15.2) % for T2-TSE, 53.6 (±11.46) for T2-CAT, 70.1 (±15.5) % for PD-TSE and 37.4 (±14.5) % for PD-CAT (cf. Fig. 6). Mean SAR differences between TSE and CAT were 29.0 (±5.7) % for the T2-contrast and 32.7 (±21.9) % for the PD-contrast (cf. Table 2). The SAR reduction of CAT compared to TSE sequences was significant for both, the T2- and PD-contrast (*paired t-tests*, p<0.001).

**Table 2:** Specific absorption rates (SAR).

	SARmean ± SD[% ]§		SARmean ± SD[W/kg ]		Δ SAR _TSE – CAT_mean ± SD[% ]§		Δ SAR _TSE – CAT_mean ± SD[W/kg ]	
**T2-TSE**	82.6	15.2	2.64	0.49	29.0*	5.7	0.93*	0.18
**T2-CAT**	53.6	11.46	1.72	0.37				
**PD-TSE**	70.1	15.5	2.24	0.48	32.7*	21.9	1.05*	0.70
**PD-CAT**	37.4	14.5	1.20	0.50				

§ 100% corresponding to SAR limit of 3.2 W/kg for head examinations according to the IEC regulation.

*Difference significant (*paired t-tests*; α = 0.05; p<0.001).

For T2-TSE, SAR values exceeded 90% in n = 15 out of 40 patients (37.5%). For PD-TSE, SAR values exceeded 90% in n = 3 out of 40 patients (7.5%). On the contrary, T2- and PD-CAT did not result in SAR values ≥90% in any of the patients. For TSE, the turbo factor (TF) had to be reduced from 13 to 9 in n = 4 out of 40 patients (10%) to enable MR measurements. Otherwise, the SAR limits were exceeded. CAT did not exceed SAR limits in these cases even without this adjustment of the TF. However, the TF was also set to 9 for T2−/PD-CAT in these instances to ensure full equivalence and comparability with TSE.

### Acoustic Noise Measurements

**Figure 7:**
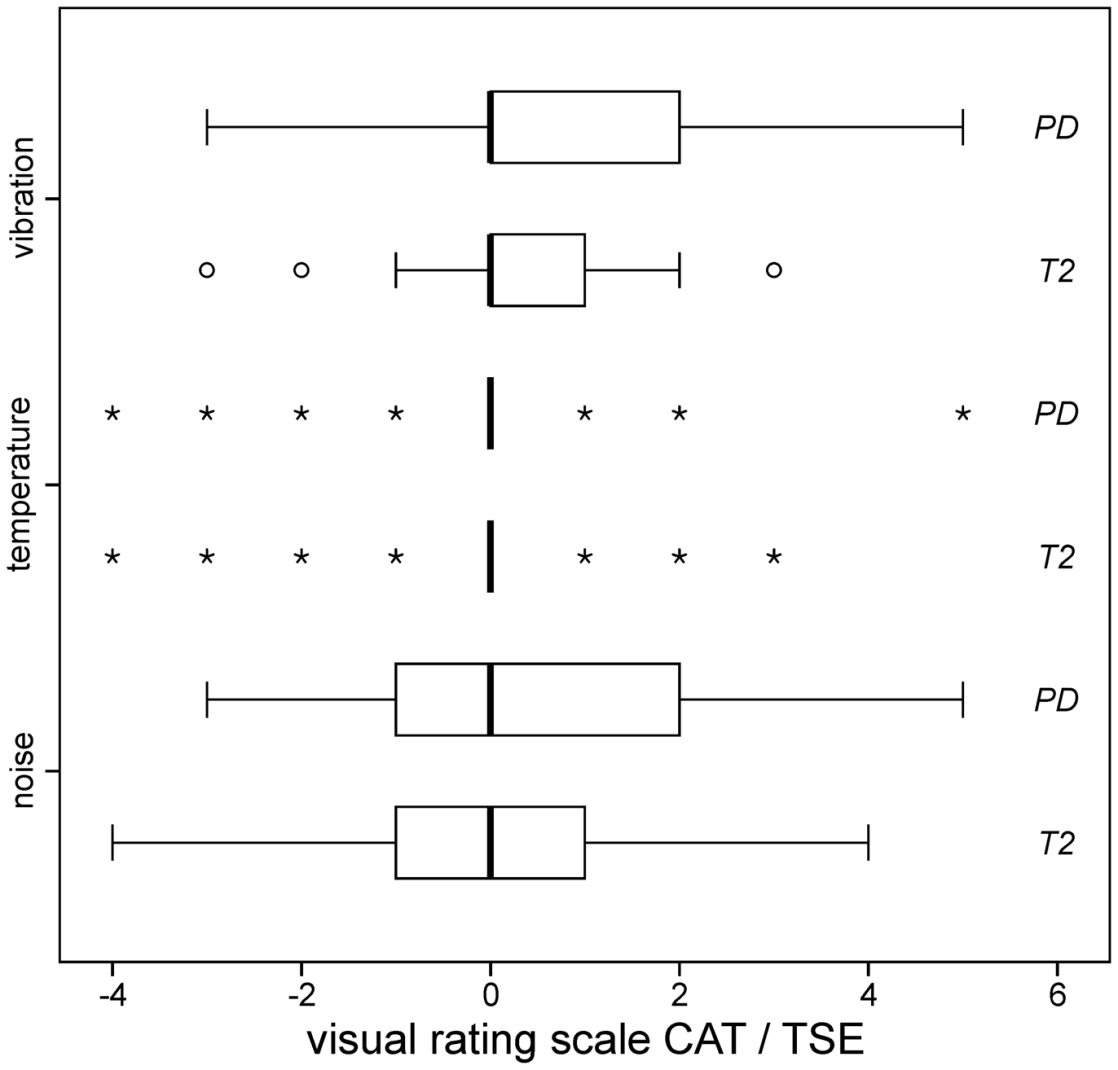
Measured noise levels. There was no difference in average SPL [dB] of T2-TSE and T2-CAT sequences. Average noise levels of PD-TSE exceeded those of PD-CAT imaging slightly (by 3.8±2.2 dB; p<0.01). (*box*: upper and lower quartiles, *thick black line*: median, *whiskers*: most extreme values of the interquartile range, *circle*: outlier).

The average SPL was 74.3 (±6.3) dB for T2-TSE, 74.0 (±6.7) dB for T2-CAT, 76.9 (±6.3) for PD-TSE and 73.1 (±6.8) dB for PD-CAT (cf. Fig. 7). There was no difference between the average SPL of axial T2-TSE and CAT (*paired t-test*, p = 0.45) while average SPL of sagittal PD-TSE recordings were minimally but consistently above those of the CAT counterparts, i.e. by 3.8 (±2.2) dB (p<0.01). This was verified in a phantom where 76 dB were measured for sagittal as well as axial PD-TSE and 72 dB for sagittal as well as axial PD-CAT. No differences were recorded for axial and sagittal T2-TSE vs. CAT (75 dB each). Thus, disabling the T2-contrast (and not sagittal slice angulation) turned PD-TSE slightly louder than CAT but this was below subjective detection levels according to the ratings collected (see below).

Sound waves and frequency spectra of exemplary T2-TSE, CAT (λ = 0.5) and a pure EPI (using λ = 0.0) recording are depicted in Figure 2. Although average SPL is comparable for CAT and TSE, peak SPL is increased by approximately 25% in CAT compared to TSE due to its EPI module (Fig. 2, top). The fundamental EPI read-out frequency peak is given by the reciprocal of twice the echo spacing (i.e., around 190 Hz in our recordings) and increases the higher the EPI part is in the sequence (i.e., the lower its CAT factor λ; Fig. 2, bottom).

### Subjective Heating, Acoustic Noise and Vibration Ratings

**Figure 8:**
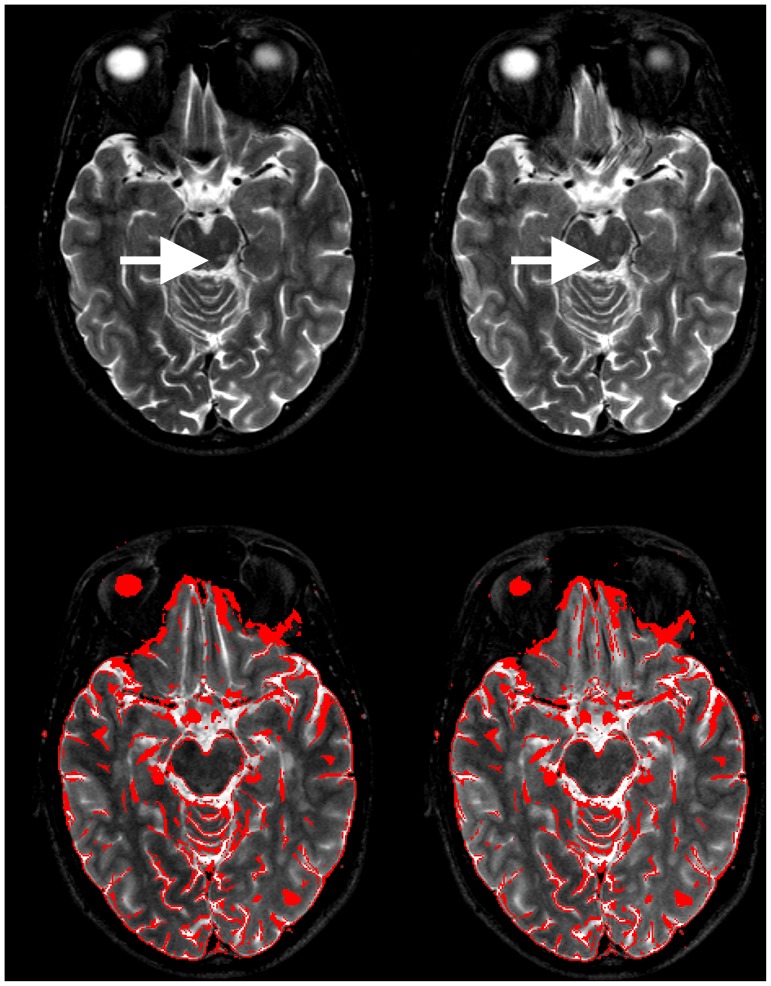
Subjective ratings. Rated sensations of RF-induced heating (*top row*), acoustic noise (*middle row*) and scanning vibrations (*bottom row*) for CAT compared to TSE (cf. Fig. 3). For the temperature ratings, only n = 7 *asterisks* for PD and T2 scanning are displayed because just this few patients noticed temperature differences between CAT and TSE while the rest (n = 33 out of 40 patients; 82.5%) perceived zero difference. None of the ratings revealed significant differences between CAT and TSE, indicating that CAT is no more uncomfortable than TSE scanning. (*box*: upper and lower quartiles, *thick black line*: median, *whiskers*: most extreme values of the interquartile range, *circle*: outlier, *asterisk*: extreme value).

For both contrasts (T2 and PD), statistical analysis demonstrated no differences in the subjective sensation of heating (T2: p = 0.80, PD: p = 0.60), acoustic noise (T2: p = 0.85, PD: p = 0.26) and scanning vibrations (T2: p = 0.21, PD: p = 0.08) between TSE and CAT (*one sample two-tailed t-tests*) (cf. Fig. 8). Thus, CAT was perceived no more uncomfortable than TSE scanning.

### Peripheral Nerve Stimulation

One of the n = 40 patients (2.5%) suffered from peripheral nerve stimulation in one leg during the axial T2-CAT and from subtle muscle twitches in the entire body during sagittal PD-CAT. Both caused the patient to press the alarm button and interrupt the exam. The patient allowed us to repeat the MR scans at doubled gradient ramp times of the EPI read-out (Ramp Time Extension Factor RTEF = 2.0). This eliminated the symptoms of peripheral nerve stimulation and decreased the average SPL of CAT marginally (by <1 dB). The RTEF was implemented as adjustable parameter in the graphical user interface (GUI) of our CAT pulse sequence.

### Image Noise, Artifacts and Distortions

Spatial noise ratios of TSE to CAT were 0.82 (T2) vs. 0.88 (PD) for the images shown in Figure 4 and 0.81 (T2) vs. 0.87 (PD; images not shown) for the patient depicted in Figure 5. The standard deviation of the noise was increased by a factor of max. 1.22 for CAT compared to TSE at the selected λ-values. T2-CAT revealed minimal artifacts and geometric distortions in the phase-encoding direction compared to TSE, especially at the base of the skull in proximity to the paranasal sinuses and the temporal bone (Fig. 5, top). Both neuroradiological readers did not consider these diagnostically relevant. Above this level, CAT and TSE were almost perfectly co-registered by rigid-body, linear transformations of 6 DoF (cf. Fig. 5, bottom). The RMS deviation did never exceed the edge length of the in-plane resolution (e.g. the RMS deviation amounted to 1.6e^−6^ mm for CAT registered to TSE in Fig. 5). Additional areas of signal loss were not observed, and for PD-CAT geometric distortions were below the visual detection limit (even after the inverse consistent rigid-body registration using mri_robust_register; see *Materials and Methods/Image Processing* section above).

## Discussion

### MS Lesion Detection

In this study, MS lesion detection of CAT and TSE based PD-/T2-images were compared by two experienced, board-certificated neuroradiologists. The results provide the first evidence that CAT is indeed diagnostically equivalent to TSE in characterizing the MS lesion load which is essential given that the SNR achieved by CAT is known to be slightly reduced compared to TSE [7]. Furthermore, our data support the notion that CAT imaging can be extended to other clinical applications in order to reduce the RF burden and to facilitate standardized MRI below the SAR limits (cf. Information S1, *Image noise, artifacts and distortions* section, last paragraph). PD-/T2-CAT as used here will be the basis for further successfully implementing a robust FLAIR-CAT pulse sequence which requires an additional magnetization preparation scheme with an additional inversion pulse. Programming and evaluation of a FLAIR-CAT pulse sequence is currently under active development and in progress. Regardless of its future availability, PD/T2-CAT will be valuable because this provides the preferred contrast at the infratentorial level where FLAIR is susceptible to increased artifacts due to CSF pulsation, in particular.

### Specific Absorption Rate (SAR) and Protocol Standardization

As has been demonstrated for the field strength of 3 Tesla, the CAT approach helps to lower the global SAR when compared to TSE sequences [7]. In this study, SAR values of the CAT sequence were 29% (T2-CAT) and 33% (PD-CAT) below those of the TSE technique (with 100% corresponding to SAR limit of 3.2 W/kg for head examinations according to the effective IEC regulation). This reduced RF burden is especially relevant for clinical routine imaging at high magnetic fields of 3 Tesla and beyond. It may be crucial in order to translate MRI at 7 Tesla into clinical applications, in particular. However, for field strengths of 7 Tesla and above, the reduced radio-frequency (RF) wavelength may lead to undesired focusing of RF-fields and in some regions the peak local SAR_10 g_ (i.e. the local SAR when averaged over 10 g of tissue) may exceed IEC regulatory limits [13] before the global SAR limits are reached. Assessing local SAR_10 g_ values *in vivo* at high fields is a very challenging task, especially when multi-channel transmit systems or RF shimming methods are applied. Current methods for local SAR_10 g_ prediction mainly rely on extensive numerical RF-field simulations for different imaging conditions [19], [20]. The CAT approach decreases the global SAR (and thus also the local SAR_10 g_) by reducing the number of RF pulses for a given echo-train length. In cases where the local SAR_10 g_ may still exceed the safety limits in ultra-high field applications, the CAT sequence can be combined with other techniques for further SAR reduction such as RF-shimming [20], low refocusing flip angles [21] or parallel MRI.

Alternatively, the advantage of reduced SAR in CAT imaging may directly be transferred to an increased number of recorded slices, higher spatial image resolution and/or reduced acquisition times which could possibly reduce motion artifacts (example values are provided in the Information S2, *Number of slices, image resolution and acquisition time with CAT* section.

Moreover, CAT imaging allows for identical sequence parameters within and across patient samples because SAR safety limits are less likely to be exceeded. In our case, 10% of the patients could not have been scanned by TSE at the originally chosen parameter settings while CAT would not have exceeded the SAR limits even without lowering the turbo factor. According to our experience, this is a realistic estimate of unwanted adjustments necessary for clinical scanning protocols and standardized trials conducted at high-field MRI. CAT can avoid such adjustments and thereby facilitate standard operating procedures and protocol settings in clinical trials and other scientific investigations.

### Subjective Heating, Acoustic Noise and Vibration Ratings

Although CAT reduced RF exposure and SAR values consistently, patients did not notice any significant temperature difference between CAT and TSE scanning. This can be explained by the conservative SAR limit of 3.2 W/kg (averaged over the head for cranial exams) which effectively prevents RF-induced heating in most patients. At more liberal SAR limits, we would expect more patients to experience heat sensations upon TSE than during CAT scans.

Given that EPI is among the loudest MRI pulse sequences due to its rapid oscillating read-out gradient switching, we also expected peak SPLs of CAT to exceed those of TSE. Higher EPI fractions (i.e., lower λ values) indeed introduce higher peak SPLs and increasing fundamental frequency peaks at the EPI read-out frequency (i.e., the reciprocal of twice the echo spacing) [9], [10] (Fig. 2, bottom). Averaged SPLs, however, of T2-CAT do not exceed those of TSE when recorded over at least one repetition cycle (Figs. 2 and 7, top). For PD-weighted imaging, average SPLs were even slightly (3.8 dB) above those of CAT. This was shown not to depend on the slice angulation but on the PD-contrast. It can be explained by the following: By measuring from the k-space centre to its outer parts, PD-CAT does in fact avoid the higher gradient switches of PD-TSE and T2-CAT. At approximately the middle of the PD-CAT recordings, only relatively small blips are used. PD-TSE and T2-CAT, on the other hand, both start with TSE in the outer k-space where increased gradient amplitudes are necessary. Thus, the fact that average SPLs of PD-TSE are above those of CAT is due to the k-space trajectory and the limited gradients used in PD-CAT.

Overall, patients sensed no differences between TSE and CAT sequences with respect to temperature, acoustic noise level and vibrations. The latter is relevant because aside from acoustic vibrations of higher frequencies EPI can also introduce uncomfortable mechanical vibrations of lower frequencies (e.g., related to the number of slices read out per TR in 2D sequences), even though this has been primarily observed in diffusion-weighted EPI [22]. Taken together, CAT scanning was not accompanied by higher levels of discomfort than TSE according to the subjective judgements of our patients.

### Peripheral Nerve Stimulation

CAT is more prone than TSE to induce symptoms and signs of peripheral nerve stimulation due to the rapid alternation of EPI read-out gradient switches. Muscle twitches during CAT imaging were indeed provoked in one of our patients. Extension of the ramp time of the read-out in the EPI module of CAT is able to avoid such stimulations (cf. Information S3, *Peripheral nerve stimulation* section).

### Image Noise, Artifacts and Distortions

It has been demonstrated previously that CAT is of reduced SNR compared to TSE [7]. Upon close visual inspection, a slight blurring especially of small MS lesions and somewhat increased noise levels may be noted in CAT images (cf. Fig. 5). Theoretically, this may identify the underlying MR sequence (CAT as opposed to TSE) which would, in turn, impede the process of blinded reading CAT vs. TSE images. However, as these were read in random order and given that every MS lesion identified on TSE was also detected on CAT even when CAT was read first, we do not consider this to be a detrimental confound of our study.

The reduced SNR of CAT did not impede equivalence of CAT and TSE because lesion count and not lesion volume is considered diagnostic by all current criteria for MS [12], [23]–[26].

### Conclusions

CAT is diagnostically equivalent to TSE imaging for MS lesion detection but reduces electromagnetic RF energy exposure. Thereby, CAT can overcome prevailing problems of strict SAR limits which we are increasingly facing with more high-field MR systems being installed for clinical neuroimaging.

There is no evidence indicating that CAT would be different from TSE for other clinical neuroimaging questions than MS lesion load. For future applications, in particular the neuroimaging of MS, fluid attenuation inversion recovery (FLAIR) sequences based on CAT remain to be developed and evaluated.

## Supporting Information

Information S1 **Image noise, artifacts and distortions.**
(DOC)

Information S2 **Number of slices, image resolution and acquisition time with CAT.**
(DOC)

Information S3 **Peripheral nerve stimulation.**
(DOC)
