# Correction: Combined Acquisition Technique (CAT) for Neuroimaging of Multiple Sclerosis at Low Specific Absorption Rates (SAR)

**DOI:** 10.1371/journal.pone.0094439

**Published:** 2014-04-04

**Authors:** The PLOS ONE Staff

The legends for Figures 2-8 were switched. The authors have provided corrected legends here.

**Figure 2:**
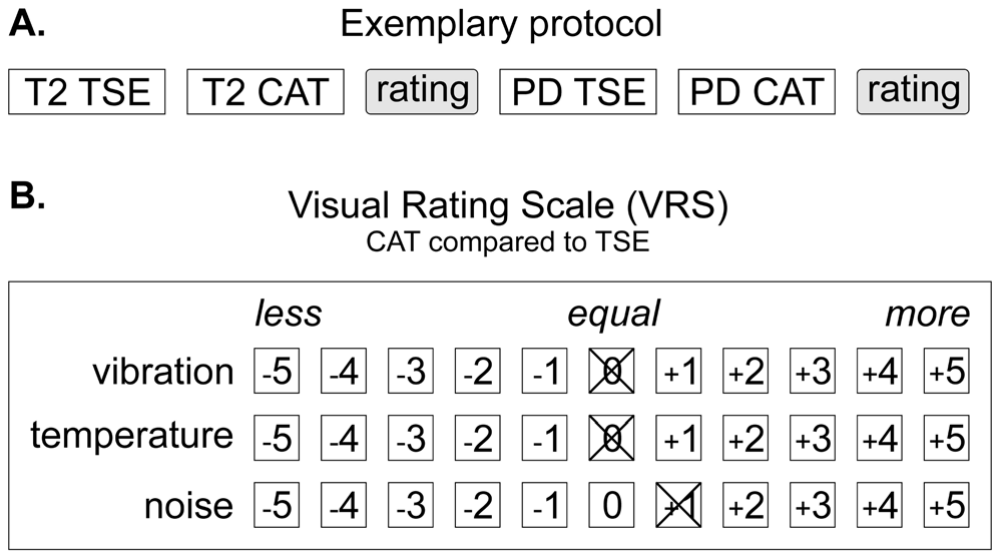
Measurement protocol and Visual Rating Scale (VRS). The order of CAT and TSE sequences was varied by rotation according to a Latin square. Upon measurement of a CAT/TSE double (**A)** the patient rated the two sequences in comparison to each other. Ratings scored sensations of temperature (RF-induced heating), acoustic noise and scan vibrations (**B**). Negative VRS values indicate less heating, acoustic noise and scan vibrations during CAT vs. TSE imaging, positive values indicate that higher temperatures, acoustic noise and vibration levels were perceived during CAT vs. TSE imaging while zero refers to no subjective difference between the CAT and TSE.

**Figure 3:**
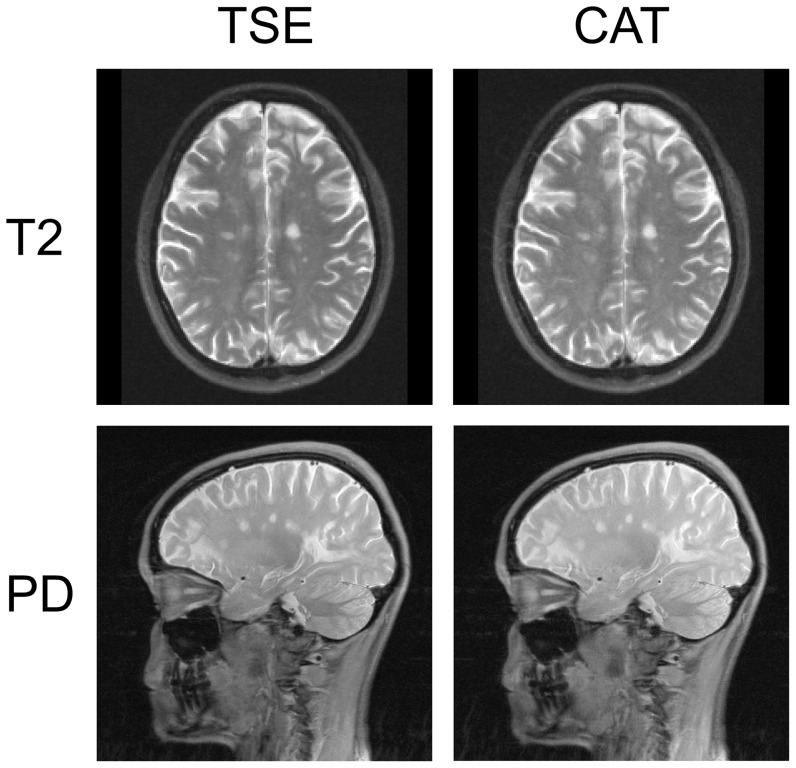
TSE and CAT brain images in Multiple Sclerosis (MS). Exemplary T2- (upper row) and PD-weighted images acquired by TSE (*left column*) and CAT (*right column*) sequences are shown. The data from this representative patient illustrate, along with those from another in Fig. 5, the diagnostic equivalence of both MR techniques: Every lesion picked up on the TSE image is detected on the CAT image as well. Minimally reduced SNR of CAT compared to TSE which has previously been quantified [7] is noticeable upon close visual inspection but does not impede diagnostic accuracy (spatial noise ratio of TSE to CAT was 0.82 for T2- and 0.88 for PD-weighted images).

**Figure 4:**
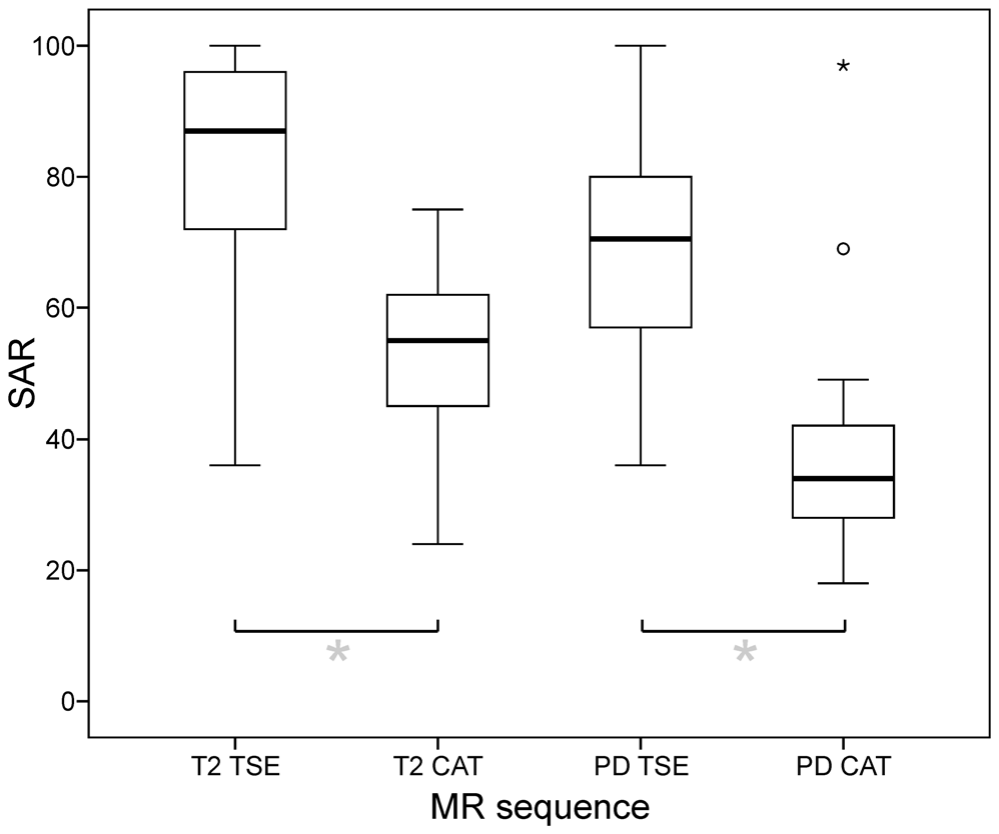
Specific absorption rate (SAR) of TSE and CAT sequences. Relative SAR reductions of CAT compared to TSE sequences was significant for both, the T2- (29.0 ± 5.7 %; p < 0.001) and the PD -contrast (32.7 ± 21.8 %; p < 0.001). (*box*: upper and lower quartiles, *thick black line*: median, *whiskers*: most extreme values of the interquartile range, *circle*: outlier, *asterisk*: extreme value; SAR values expressed as percentages of the effective SAR limit of 3.2 W/kg for head examinations according to IEC).

**Figure 5:**
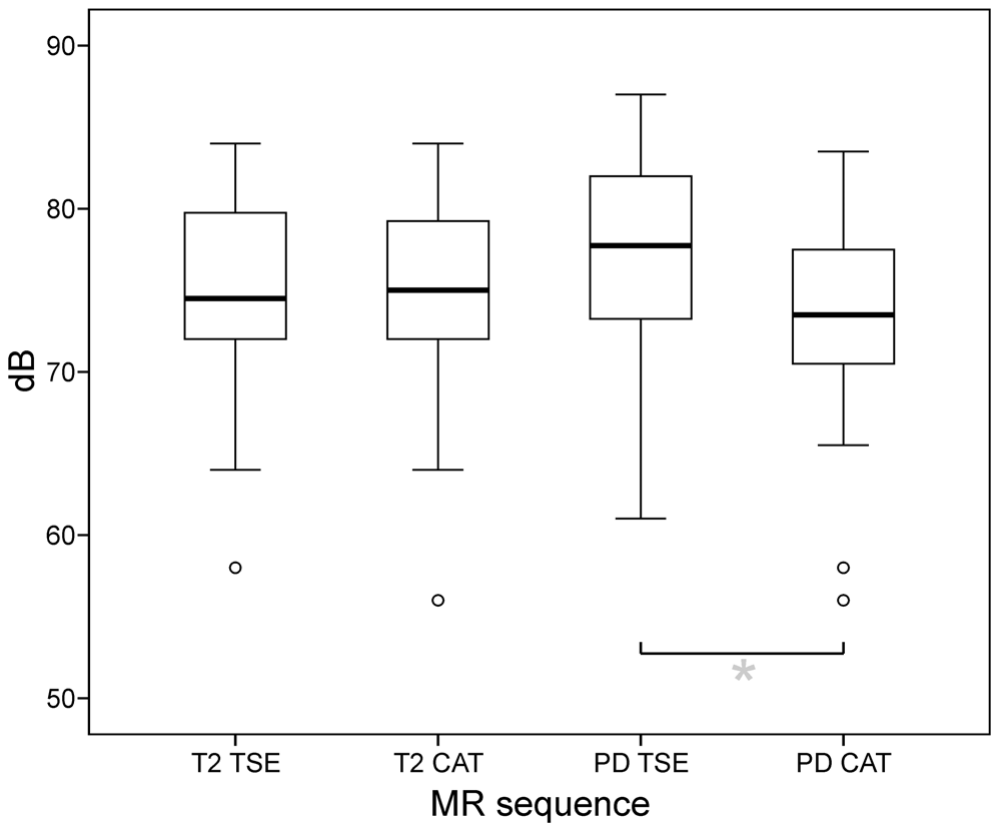
Measured noise levels. There was no difference in average SPL [dB] of T2-TSE and T2-CAT sequences. Average noise levels of PD-TSE exceeded those of PD-CAT imaging slightly (by 3.8 ± 2.2dB; p < 0.01). (*box*: upper and lower quartiles, *thick black line*: median, *whiskers*: most extreme values of the interquartile range, *circle*: outlier)

**Figure 6:**
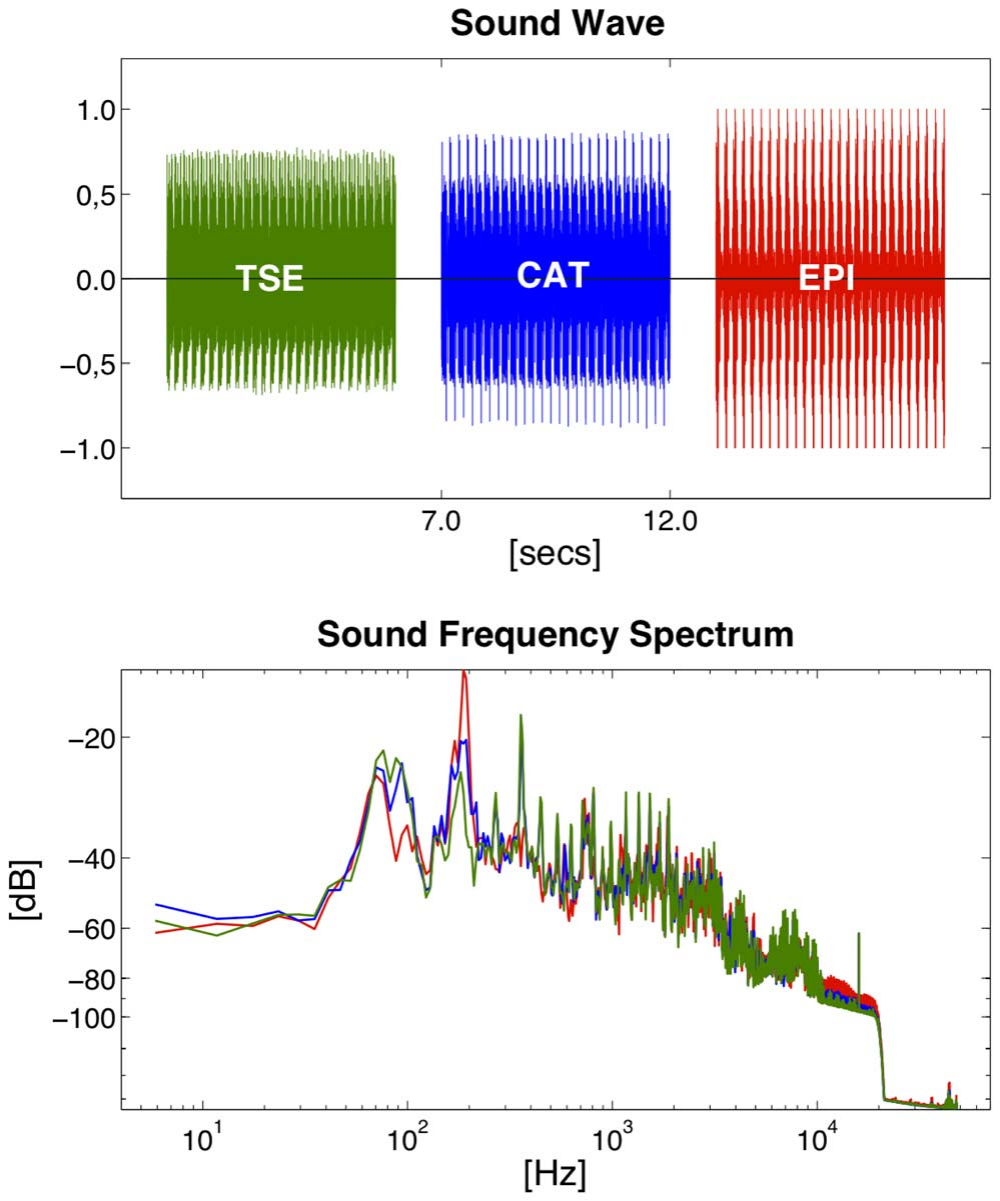
Sound waves and frequency spectra for T2-weighted TSE, CAT (λ  =  0.5) and pure EPI. Peak SPLs increase the higher the EPI proportion (i.e., the lower the CAT factor λ) but average SPLs of CAT at λ  = 0.5 and TSE are comparable (*top*). EPI read-outs introduce a fundamental frequency peak at the reciprocal of twice the echo spacing (here: ESP  =  2.6 ms/FFT peak  = 192 Hz) which increases the higher the EPI proportion (i.e., the lower λ is set; *bottom*).

**Figure 7:**
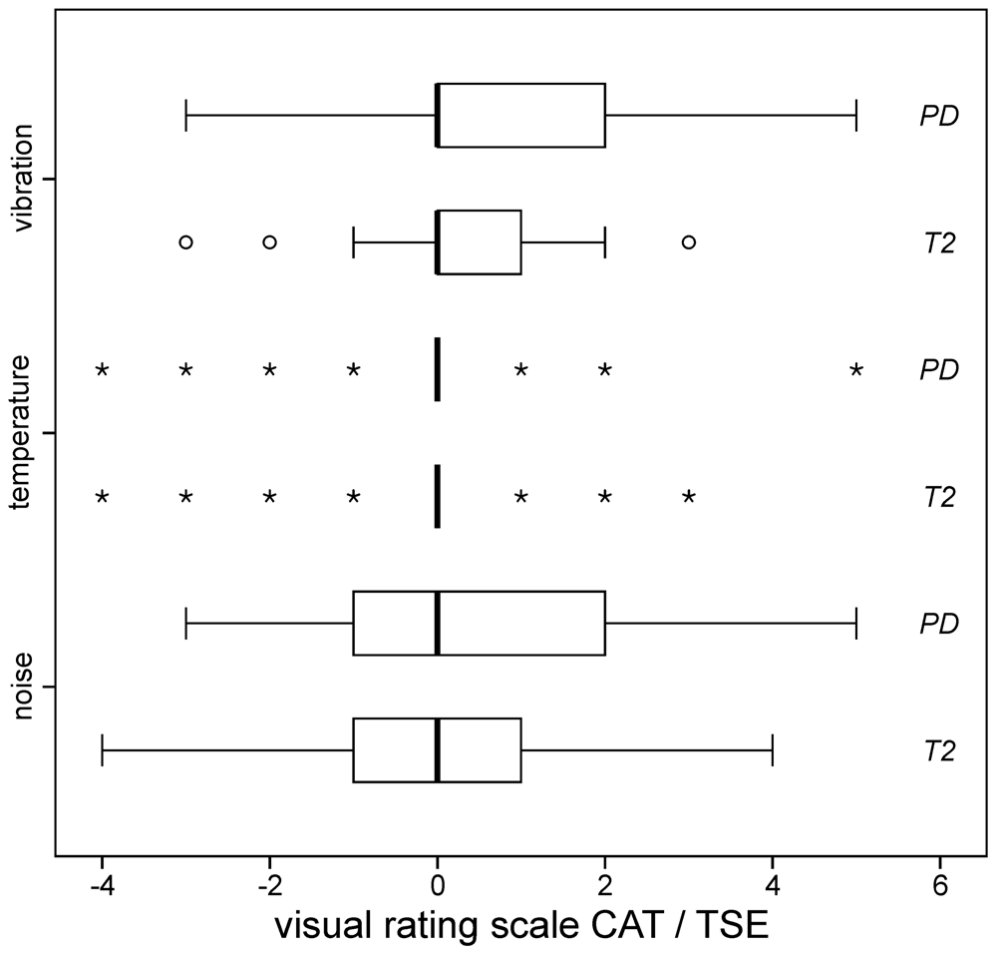
Subjective ratings. Rated sensations of RF-induced heating (*top row*), acoustic noise (*middle row*) and scanning vibrations (*bottom row*) for CAT compared to TSE (cf. Fig. 3). For the temperature ratings, only n  =  7 *asterisks* for PD and T2 scanning are displayed because just this few patients noticed temperature differences between CAT and TSE while the rest (n  =  33 out of 40 patients; 82.5 %) perceived zero difference. None of the ratings revealed significant differences between CAT and TSE, indicating that CAT is no more uncomfortable than TSE scanning. (*box*: upper and lower quartiles, *thick black line*: median, *whiskers*: most extreme values of the interquartile range, *circle*: outlier, *asterisk*: extreme value).

**Figure 8:**
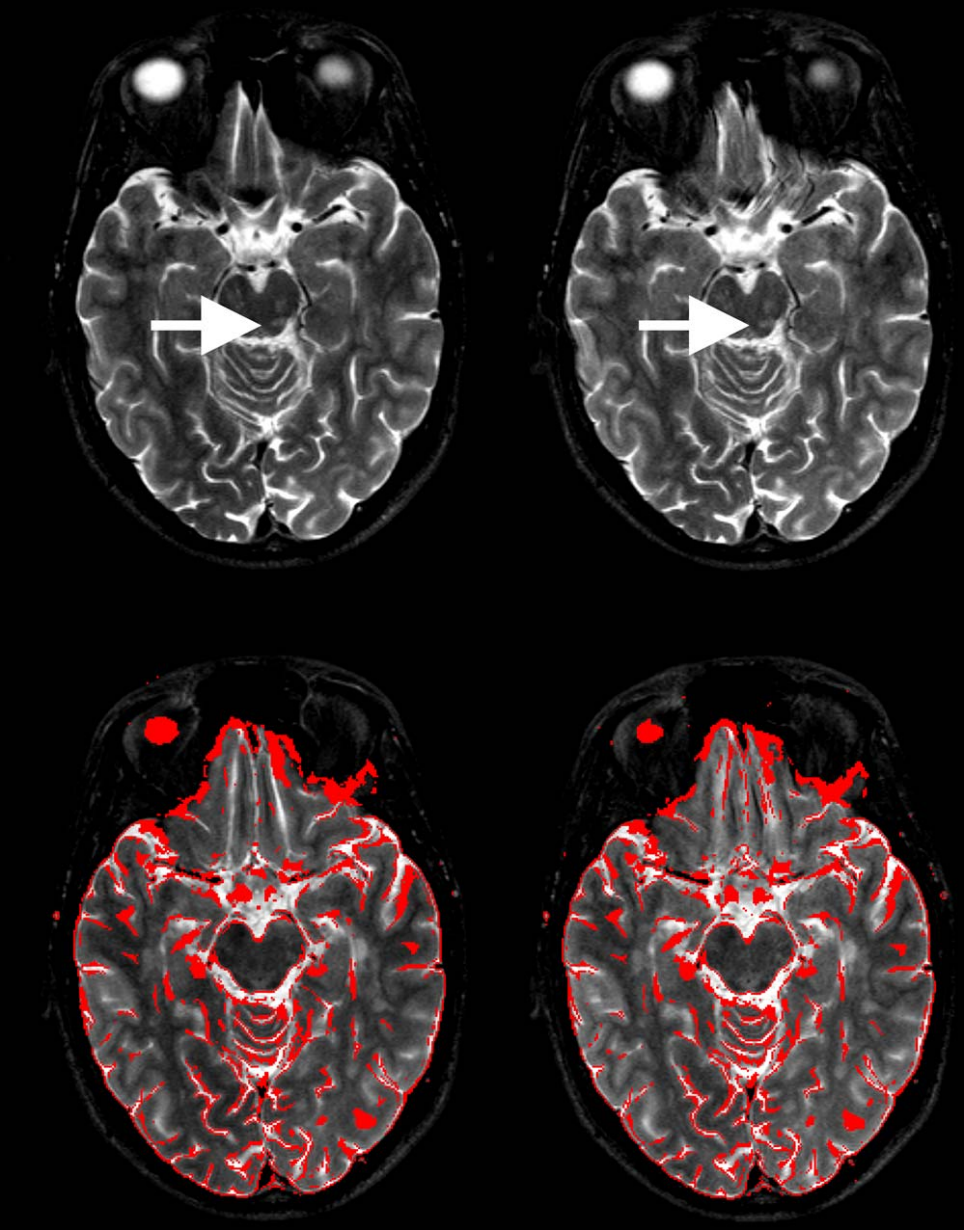
Image artifacts and distortions: CAT artifacts and distortions in phase-encoding direction (here from right to left, R>>L) as detected at the skull base level. The straight gyrus and olfactory sulcus are slightly displaced leftwards in CAT (*top right*; depending on blip polarity, cf. [7]) compared to TSE (*top left*). Otherwise, CAT contours (red outlines in *lower left*) overlay almost perfectly with TSE (*lower left*) and vice versa (*lower right*) upon CAT/TSE co-registration (RMS deviation ≤ 1.6e^-6^ mm). Artifacts did not significantly interfere with MS lesion detection. Even tiny multiple T2-hyperintense demyelinations (*arrows*) are well visualized despite minimal blurring (at a spatial noise ratio TSE/CAT of 0.81 in this case).
