# Supplementary material for: Combined Acquisition Technique (CAT) for Neuroimaging of Multiple Sclerosis at Low Specific Absorption Rates (SAR)
Source: PLoS One. 2014 Mar 7;9(3):e91030. doi: 10.1371/journal.pone.0091030 (PMC3946656; doi:10.1371/journal.pone.0091030)
Supplement: Information S1 — Image noise, artifacts and distortions. (DOC) [file pone.0091030.s001.doc]

**SUPPorting Information SI 1**

**Discussion**

**Image noise, artifacts and distortions**

It has been demonstrated previously that CAT is of reduced SNR than TSE . In the most simple form, SNR can be estimated dividing the mean signal intensity of the scanned object (brain) divided by the standard deviation outside the object and multiplied by a correction factor . For recordings with multi-channel receiver coils (12-channel phased array head coil used in our investigation), parallel acquisition techniques (like GRAPPA, not used in the current study) and in the presence of structured, non-Gaussian, spatial artifacts (cf. Fig. 5) this approach would not be accurate. In these situations, other methods employing different ROI samples or multiple replica measurements, for example, are required to measure SNR correctly . These methods require additional noise measurements. Therefore, we simply obtained spatial noise ratios of TSE to CAT for exemplary images.

According to these values, the standard deviation of the noise is increased by a factor of no more than 1.25 for CAT at the λ-values chosen. This estimate corresponds to the theoretical and empirical predictions . However, spatial noise of CAT was less increased compared to TSE for PD-weighted imaging. This may be related to the bandwidth chosen and would effectively increase the SNR in PD-weighted CAT above the expected values (cf. equation 6 in ). Mean signal and SNR may be further enhanced due to the fact that the EPI signal does not decay as much as the TSE signal (in areas where T2* approximately equals T2), simply because the EPI read-out proceeds much faster at its shorter ESP than the TSE read-out. This increases the signal particularly of the later echoes for CAT. Thus, CAT has more spatial noise than TSE but the corresponding SNR loss will be below 20 % for the given parameter settings and was hardly recognizable by eye given that SNR at 3 Tesla (and above) is rather luxurious to begin with anyway (Figs. 4 and 5). Upon close visual inspection, a slight blurring especially of small MS lesions may be noted in CAT images (Fig. 5) which is presumably related to the reduced SNR. This could, in theory, exert an impact on automatic and even manual lesion volume measurements. Such potential effect is likely to be very small. At this point, we did not examine the possibility further because lesion count and not lesion volume is considered diagnostic by all current criteria for MS and because both raters considered general lesion conspicuity to be equal on CAT and TSE.

In addition to the expense of some SNR, CAT imaging is accompanied by a few artifacts and minimal distortions in the phase-encoding direction (Fig. 5). These are primarily observed at the skull base level in proximity to air / soft tissue boundaries. Considering the actual echo spacing, which is high compared to pure EPI sequences, distortions were rather insignificant. Overall, SNR reduction, artifacts and geometric distortions of CAT did not impede diagnostic information and accuracy in the clinical setting.

Flow void in vessels (Figs. 4 & 5) and in the aquaeductus mesencephali (Fig. 5, bottom), for example, is equally well depicted on TSE and CAT. Thus, loss of flow void (such as in cases of internal carotid artery occlusion or sinus thrombosis) and increased flow void (such as in sufficient third ventriculostomia), for example, can be expected to be equally well visualized on CAT compared to TSE.

**references**

1. Choli M, Blaimer M, Breuer FA, Ehses P, Speck O, et al. (2013) Combined acquisition technique (CAT) for high-field neuroimaging with reduced RF power. MAGMA 26: 411-418.

2. Firbank MJ, Harrison RM, Williams ED, Coulthard A (2000) Quality assurance for MRI: practical experience. Br J Radiol 73: 376-383.

3. Robson PM, Grant AK, Madhuranthakam AJ, Lattanzi R, Sodickson DK, et al. (2008) Comprehensive quantification of signal-to-noise ratio and g-factor for image-based and k-space-based parallel imaging reconstructions. Magn Reson Med 60: 895-907.

4. Kellman P, McVeigh ER (2005) Image reconstruction in SNR units: a general method for SNR measurement. Magn Reson Med 54: 1439-1447.

5. Choli M, Jakob PM, Loeffler RB, Hillenbrand CM (2010) Mixed-bandwidth acquisitions: signal-to-noise ratio and signal-to-noise efficiency. J Magn Reson Imaging 32: 997-1002.

6. Filippi M, Rocca MA (2011) MR imaging of multiple sclerosis. Radiology 259: 659-681.

7. Polman CH, Reingold SC, Edan G, Filippi M, Hartung HP, et al. (2005) Diagnostic criteria for multiple sclerosis: 2005 revisions to the "McDonald Criteria". Ann Neurol 58: 840-846.

8. Swanton JK, Fernando K, Dalton CM, Miszkiel KA, Thompson AJ, et al. (2006) Modification of MRI criteria for multiple sclerosis in patients with clinically isolated syndromes. J Neurol Neurosurg Psychiatry 77: 830-833.

9. Rovira A, Swanton J, Tintore M, Huerga E, Barkhof F, et al. (2009) A single, early magnetic resonance imaging study in the diagnosis of multiple sclerosis. Arch Neurol 66: 587-592.

10. Montalban X, Tintore M, Swanton J, Barkhof F, Fazekas F, et al. (2010) MRI criteria for MS in patients with clinically isolated syndromes. Neurology 74: 427-434.
