# Supplementary material for: Combined Acquisition Technique (CAT) for Neuroimaging of Multiple Sclerosis at Low Specific Absorption Rates (SAR)
Source: PLoS One. 2014 Mar 7;9(3):e91030. doi: 10.1371/journal.pone.0091030 (PMC3946656; doi:10.1371/journal.pone.0091030)
Supplement: Information S2 — Number of slices, image resolution and acquisition time with CAT. (DOC) [file pone.0091030.s002.doc]

**SUPPorting Information SI 2**

**Discussion**

**Number of slices, image resolution and acquisition time with CAT**

At the given parameter settings CAT would allow to record 11 more axial T2-weighted slices and 18 more sagittal PD-weighted slices than TSE. Similarly, the maximal in-plane resolution in the phase encoding direction would be 0.6 mm (or 394 phase encoding steps) for TSE vs. 0.4 mm (or 550 phase encoding steps) for CAT. I.e., CAT can be used to increase the number of slices and / or the in-plane spatial resolution by least one fourth compared to TSE.

For the tested pulse sequences and their given parameter settings, the minimal TR (TA) would be 6120 ms (2:02 mins) for T2-CAT, 7760 ms (2:35 mins) for T2-TSE, 3410 ms (1:29 mins) for PD-CAT and 4840 ms (2:06 mins) for PD-TSE. Thus, CAT may also be used to reduce the time needed for image acquisition, i.e. to scan faster. Note that shortening the TR (TA) also increases the relative RF energy deposition which may then exceed the SAR limit. This, however, would affect TSE before CAT.
