# Supplementary material for: Combined Acquisition Technique (CAT) for Neuroimaging of Multiple Sclerosis at Low Specific Absorption Rates (SAR)
Source: PLoS One. 2014 Mar 7;9(3):e91030. doi: 10.1371/journal.pone.0091030 (PMC3946656; doi:10.1371/journal.pone.0091030)
Supplement: Information S3 — Peripheral nerve stimulation. (DOC) [file pone.0091030.s003.doc]

**SUPPorting Information SI 3**

**Discussion**

**Peripheral nerve stimulation**

In addition to their associated sound characteristics, rapidly oscillating EPI read-out gradients are prone to increase the incidence of peripheral nerve stimulations in susceptible patients during CAT. Muscle twitches during CAT imaging were indeed provoked in one of our patients despite adherence to the effective safety limits. Extension of the ramp time of the read-out in the EPI module of CAT is able to avoid such stimulations. We successfully implemented a Ramp Time Extension Factor (RTEF) in the Graphical User Interface (GUI) of our CAT protocol for precisely this purpose. In addition, our CAT-GUI now allows setting the T2-contrast using the echo time (TE) directly like in standard sequences. This simplifies the TE calculation and specification for T2-CAT which is determined by the turbo factor (TF), the CAT-factor (λ) and the echo spacing (ESP) of the TSE-module.
